# *Longistriataflava* (Boletaceae, Basidiomycota) – a new monotypic sequestrate genus and species from Brazilian Atlantic Forest

**DOI:** 10.3897/mycokeys.62.39699

**Published:** 2020-02-03

**Authors:** Marcelo A. Sulzbacher, Takamichi Orihara, Tine Grebenc, Felipe Wartchow, Matthew E. Smith, María P. Martín, Admir J. Giachini, Iuri G. Baseia

**Affiliations:** 1 Departamento de Micologia, Programa de Pós-Graduação em Biologia de Fungos, Universidade Federal de Pernambuco, Av. Nelson Chaves s/n, CEP: 50760-420, Recife, PE, Brazil Universidade Federal de Pernambuco Recife Brazil; 2 Kanagawa Prefectural Museum of Natural History, 499 Iryuda, Odawara-shi, Kanagawa 250-0031, Japan Kanagawa Prefectural Museum of Natural History Odawara Japan; 3 Slovenian Forestry Institute, Večna pot 2, SI-1000 Ljubljana, Slovenia Slovenian Forestry Institute Ljubljana Slovenia; 4 Departamento de Sistemática e Ecologia/CCEN, Universidade Federal da Paraíba, CEP: 58051-970, João Pessoa, PB, Brazil Universidade Federal da Paraíba João Pessoa Brazil; 5 Department of Plant Pathology, University of Florida, Gainesville, Florida 32611, USA University of Florida Gainesville, FL United States of America; 6 Departamento de Micologia, Real Jardín Botánico, RJB-CSIC, Plaza Murillo 2, Madrid 28014, Spain Departamento de Micologia, Real Jardín Botánico Madrid Spain; 7 Universidade Federal de Santa Catarina, Departamento de Microbiologia, Imunologia e Parasitologia, Centro de Ciências Biológicas, Campus Trindade – Setor F, CEP 88040-900, Florianópolis, SC, Brazil Universidade Federal de Santa Catarina Florianópolis Brazil; 8 Departamento de Botânica e Zoologia, Universidade Federal do Rio Grande do Norte, Campus Universitário, CEP: 59072-970, Natal, RN, Brazil Universidade Federal do Rio Grande do Norte Natal Brazil

**Keywords:** Boletales, ITS, phylogeny, sequestrate fungi, taxonomy, tropical forest.

## Abstract

A new monotypic sequestrate genus, *Longistriata* is described based on collections from the Neotropical forest of Atlantic forest in Paraíba, Northeast Brazil – an area known for its high degree of endemism. The striking features of this new fungus are the hypogeous habit, the vivid yellow peridium in mature basidiomes, broadly ellipsoid basidiospores with a distinct wall that is ornamented with longitudinal striations and lageniform cystidia with rounded apices. Phylogenetic analysis, based on LSU and *tef-1α* regions, showed that the type species, *Longistriataflava*, is phylogenetically sister to the monotypic sequestrate African genus *Mackintoshia* in Boletaceae. Together these two species formed the earliest diverging lineage in the subfamily Zangioideae. *Longistriataflava* is found in nutrient-poor white sand habitats where plants in the genera *Coccoloba* (Polygonaceae) and *Guapira* (Nyctaginaceae) are the only potential ectomycorrhizal host symbionts.

## Introduction

Fungi in the order Boletales (Agaricomycetes, Basidiomycota) comprise a morphological diverse group including agaricoid, boletoid, gasteroid, secotioid, corticioid, merulioid, hydroid and polyporoid forms (Binder and Hibbett 2006) with ectomycorrhizal (ECM), saprophytic or ligninolytic members (Kirk et al. 2008). The order is a globally distributed group of mushroom-forming fungi growing in most forest ecosystems (Chu-Chou and Grace 1983; Binder and Hibbett 2006). Despite thorough morphological (Rolland 1899; Høiland 1987; Pegler and Young 1989; Montecchi and Sarasini 2000) and phylogenetic coverage of the order Boletales (Kretzer and Bruns 1999; Binder and Bresinsky 2002; Binder and Hibbett 2006; Orihara et al. 2016a) new phylogenetically supported genera are still being discovered, particularly representatives with a sequestrate habitat (Nuhn et al. 2013; Wu et al. 2014, 2016). The sequestrate habitat has arisen in this order multiple times and a large number of sequestrate genera in Boletaeceae have been described: *Carolinigaster* M.E. Sm. & S. Cruz (Crous et al. 2018), *Chamonixia* Rolland (Binder and Bresinsky 2002; Orihara et al. 2016a), *Heliogaster* Orihara & Iwase (Orihara et al. 2010), *Kombocles* Castellano, T.W. Henkel & Dentinger (Castellano et al. 2016), *Octaviania* Vittad. (Vittadini 1831; Orihara et al. 2012), *Mackintoshia*Pacioni and Sharp (2000). *Rhodactina* Pegler and T.W.K. Young (Yang et al. 2006; Vadthanarat et al. 2018), *Rossbeevera* T. Lebel and Orihara (Lebel et al. 2012), *Royoungia* Castellano, Trappe and Malajczuk (Castellano et al. 1992), *Solioccasus* Trappe et al. (Trappe et al. 2013), *Turmalinea* Orihara and N. Maek. (Orihara et al. 2016b) and *Afrocastellanoa* M.E. Smith & Orihara (Orihara and Smith 2017).

Sequestrate Boletaceae have been described from across the globe with records from all continents except Antarctica but relatively little is known about sequestrate boletoid fungi in South America (Putzke 1994; Sulzbacher et al. 2017). Species of *Rhizopogon* Fr. & Nordholm (Fries and Nordholm 1817) and *Scleroderma* Pers. (Persoon 1801) are broadly distributed and most frequently recorded in forest plantations with introduced pines, eucalypts or pecan trees (Martín 1996; Giachini et al. 2000; Baseia and Milanez 2002; Nouhra et al. 2012; Sulzbacher et al. 2016a, 2018). However, there are relatively few citations of sequestrate taxa from native ectotrophic forests. Examples from temperate habitats include *Alpovaaustroalnicola* L.S. Domínguez in AlnusacuminataKunthssp.acuminata forests in the Yunga District of Argentina (Nouhra et al. 2005) and *Sclerodermapatagonicum* Nouhra & Hern. Caff. in Patagonian *Nothofagus* forests (Nouhra et al. 2012). Recently, undescribed taxa of sequestrate Boletaceae were cited from tropical forests in Guyana (Henkel et al. 2012; Smith et al. 2013) and formally described as *Jimtrappea* T.W. Henkel, M.E. Smith & Aime, *Castellanea* T.W. Henkel & M.E. Sm. and *Costatisporus* T.W. Henkel & M.E. Sm. (Smith et al. 2015). These new records from the Guiana Shield suggest that other unexplored tropical forests in South America may host additional diversity of sequestrate Boletales, similar to recent reports from Asia and Africa (Castellano et al. 2016; Chai et al. 2019).

In Brazil, there are numerous surveys that have documented epigeous Boletales in exotic plantations and native forests (Rick 1961; Guzmán 1970; Putzke 1994; Watling and de Meijer 1997; Baseia and Milanez 2000; Giachini et al. 2000; Baseia and Milanez 2002; Sobestiansky 2005; de Meijer 2006; Gurgel et al. 2008; Cortez et al. 2011; Magnago and Neves 2014; Barbosa-Silva and Wartchow 2017; Barbosa-Silva et al. 2017; Magnago et al. 2017a, [Bibr B35][Bibr B36], 2019). However, information related to sequestrate hypogeous fungi is scanty (Sulzbacher et al. 2016a).

As part of recent studies on ectomycorrhizal and sequestrate fungi in northeastern Brazil (Sulzbacher et al. 2013, 2017), we collected a sequestrate taxon that could not be assigned to any current species in the family Boletaceae. Here we describe and characterize the new sequestrate boletoid species in a newly erected genus *Longistriata* based on sequence analyses of the ITS, nLSU, and *TEF1* molecular markers as well as detailed analysis of morphological features. From available collections and publicly available sequences we discuss how this new species differs from all currently described genera in Boletales and we discuss the trophic mode of this new species and genus.

## Methods

### Sampling and morphological studies

**Figure 1.**
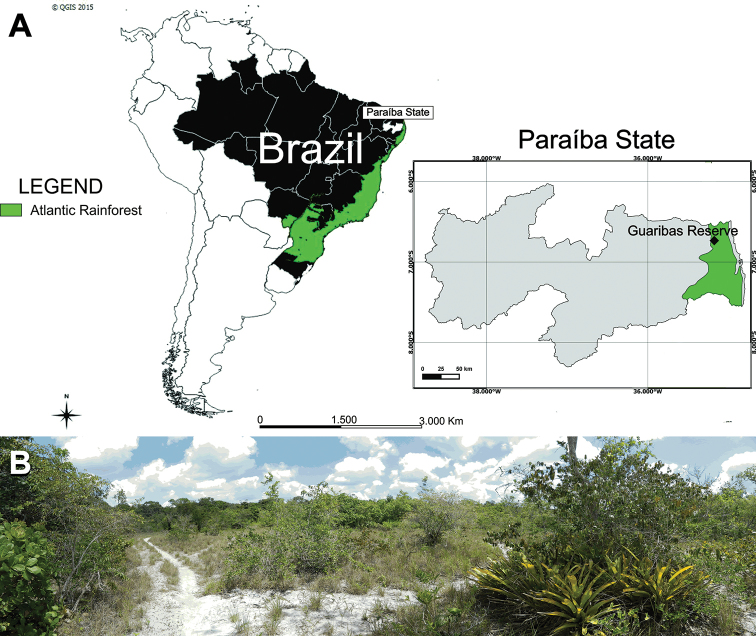
**A** Map of South America with Atlantic rainforest (in green) and magnified area of the State of Paraiba, including the location of the Guaribas Biological Reserve **B** the sampling sites at the Guaribas Biological Reserve with typical vegetation in the white sandy soil ecosystem.

Specimens were collected in survey missions targeting sequestrate fungi during the rainy seasons of 2011–2013 (Sulzbacher et al. 2016b). Sampling sites were located in forests at the Guaribas Biological Reserve, between 06°39'47"S and 06°42'57"S and 35°06'46"W’ and 35°08'00"W (Barbosa et al. 2011). This area is a protected Atlantic rainforest reserve comprising 4029 ha that is in the vicinity of the cities of Mamanguape and Rio Tinto in the state of Paraíba, Brazil (Fig. 1A). Soils are of the Tertiary sediments of the Barreiras Formation (Barbosa et al. 2011). The predominant vegetation ranges from lowland semi-deciduous forest to savanna, also known as “tabuleiro” (Fig. 1B). The dominant plant families in the Guaribas Biological Reserve are Cyperaceae, Fabaceae, Melastomataceae, Myrtaceae, Poaceae, Polygonaceae and Rubiaceae (Barbosa et al. 2011). Confirmed ectomycorrhizal host plants in this region include species of *Coccoloba* (Polygonaceae) (Bâ et al. 2014; Põlme et al. 2017) and *Guapira* (Nyctaginaceae) (Wang and Qiu 2006; Tedersoo et al. 2010a). Basidiomata were discovered using the methodology described in Castellano et al. (2004) by raking the leaf litter and topsoil. All basidiomata were photographed *in situ* and then dried in a forced-air dryer. Macro- and microscopic characters were observed with a stereomicroscope (EZ4 Leica, Leica Microsystems, Mannheim, Germany) and light microscope (Eclipse Ni Nikon, Nikon Corporation, Tokyo, Japan). Line drawings of microscopic structures were made with the aid of a drawing tube (BX41 Olympus, Olympus America Inc., Melville, NY, USA). Basidiospore data follows the methodology proposed by Tulloss et al. (1992). Measurements and statistics are based on 30 mature spores. Abbreviations include L(W) = average basidiospore length (width), Q = the length:width ratio range as determined from all measured basidiospores, and Qm = the Q value averaged from all measured basidiospores. Colors of basidiomes were observed from fresh material with color coding following Methuen Handbook of Colour (Kornerup and Wanscher 1978). The holotype is deposited at the herbarium of the Universidade Federal do Rio Grande do Norte (**UFRN**) with additional material deposited at the herbarium of the Slovenian Forestry Institute (**LJF**).

### DNA extraction, PCR amplification and sequencing

Fungal DNA was extracted from fresh specimens (UFRN-fungos 1756 and UFRN-fungos 2110) using a DNeasy Plant Mini Kit (QIAGEN) according to the manufacturer’s instructions. Partial sequences were obtained from the nuclear internal transcribed spacer (ITS) and a large subunit (nLSU) of nuclear ribosomal DNA, with the primer pairs ITS1F/ITS4 (Gardes and Bruns 1993; White et al. 1990) and LR0R/LR7 (Vilgalys and Hester 1990), respectively. Sequences were also obtained from the translation elongation factor 1-α gene (*TEF1*) with primer pair EF1-983F/EF1-1953R (Rehner and Buckley 2005). PCR reactions were performed according to Sulzbacher et al. (2016a). PCR was performed in a PTC-100 Thermocycler (MJ Research, Inc.) under the following conditions: first extension at 94 °C for 30 sec; denaturation at 94 °C for 45 sec; annealing at 55 °C (30 sec), extension at 72 °C (60 sec) for 35 cycles; and a final extension at 72 °C for 10 min. The PCR product was fractionated by electrophoresis on an 1.2% agarose gel in TBE buffer and then stained with ethidium bromide under UV light (360 nm). DNA was sequenced using a double-stranded DNA template of PCR product following the protocol supplied by Amersham Bioscience in a MegaBACE 500 (Amersham Biosciences Corp, Piscataway, NJ, USA). Newly obtained sequences were compared with homologous sequences available in the International Nucleotide Sequence Databases through BLASTn searches (Altschul et al. 1997).

### Phylogenetic analyses

Suppl. material 1: Table S1 shows the sequences of nLSU and *TEF1* that were retrieved from the International Nucleotide Sequence Databases for our analyses. Sequences were carefully selected so that the dataset included representative genera from across the Boletaceae based on Wu et al. (2016). Sequences of *Chalciporus* spp. and *Buchwaldoboletuslignicola* (Kallenb.) Pilát were used as outgroups. Sequence alignment was performed with the online version of MAFFT v. 7 (Katoh and Standley 2013) under default settings (i.e., the alignment algorithm is automatically selected from FFT-NS-1, FFT-NS-2, FFT-NS-i or L-INS-i). Subsequently, the sites with obvious alignment errors were manually adjusted in SEAVIEW v. 4. Prior to multigene analyses, we compared the neighbor joining clustering method (NJ) tree topologies between the nLSU and *TEF1* datasets on the SEAVIEW v. 4 platform. Since no major topological conflict (NJ bootstrap values ≥75%) was seen between the resulting nLSU and *TEF1* trees, we subsequently concatenated the two datasets for the multigene analyses. The *TEF1* region was partitioned by codons and introns, and best-fit likelihood models were estimated for each partition with MrModeltest v. 2.3 (Nylander 2004).

Bayesian analyses were conducted with MrBayes 3.2 (Ronquist and Huelsenbeck 2003). The SYM + G model (symmetrical nucleotide substitution model with gamma distributed rate variation among sites) was selected for nLSU and all of the codons and partitions of *TEF1*. Bayesian posterior probabilities (PP) were estimated by the Metropolis-coupled Markov chain Monte Carlo method (Geyer 1991). In the multigene (*nLSU* + *TEF1*) analysis, two parallel runs were conducted with one cold and seven heated chains each for 10M generations. The parameter for the temperature of the seven heated chains in both runs was set to 0.10. The 0.10 heating scheme was used instead of the default 0.20 setting because convergence was not achieved during preliminary runs at the 0.20 setting, probably due to Markov chains being trapped in local optima. Trees were saved to a file every 1000^th^ generation. We determined that the two runs reached convergence when the average standard deviation of split frequencies (ASDSF) was continuously lower than 0.01. The ASDSF was monitored every 5000 generations. We also verified the convergence by checking that the effective sample size (ESS) of each resulting statistic was sufficiently large (> 200). Trees obtained before reaching convergence were discarded as the burn-in, and the remaining trees were used to calculate a 50% majority consensus topology and to determine PP values for individual branches.

Maximum likelihood (ML) analyses were conducted with RAxML 8.2.10 (Stamatakis 2014). The same partitioned datasets as those for the Bayesian analyses were used so that different α-shape parameters, GTR rates (general time reversible substitution model), and empirical base frequencies could be assigned to each partition. The best-fit ML tree was estimated under the GTR+I+G models. The rapid bootstrap (BS) analysis was implemented with 1000 replicates.

## Results

The *nLSU* + *TEF1* combined dataset consisted of 85 taxa and 2,014 aligned nucleotide positions. The Bayesian inference reached convergence after 4.6M generations. We therefore discarded the first 4,600 trees in each chain, and the remaining 5,401 trees in each chain were summarized to approximate Bayesian posterior probabilities (PPs). ESS of all the model parameters were sufficiently large (>200). The total arithmetic and harmonic means of Likelihoods (lnL) were -29,498.16 and -29,562.71, respectively. In RAxML analysis the log likelihood of the ML tree was -29,121.825209.

**Figure 2.**
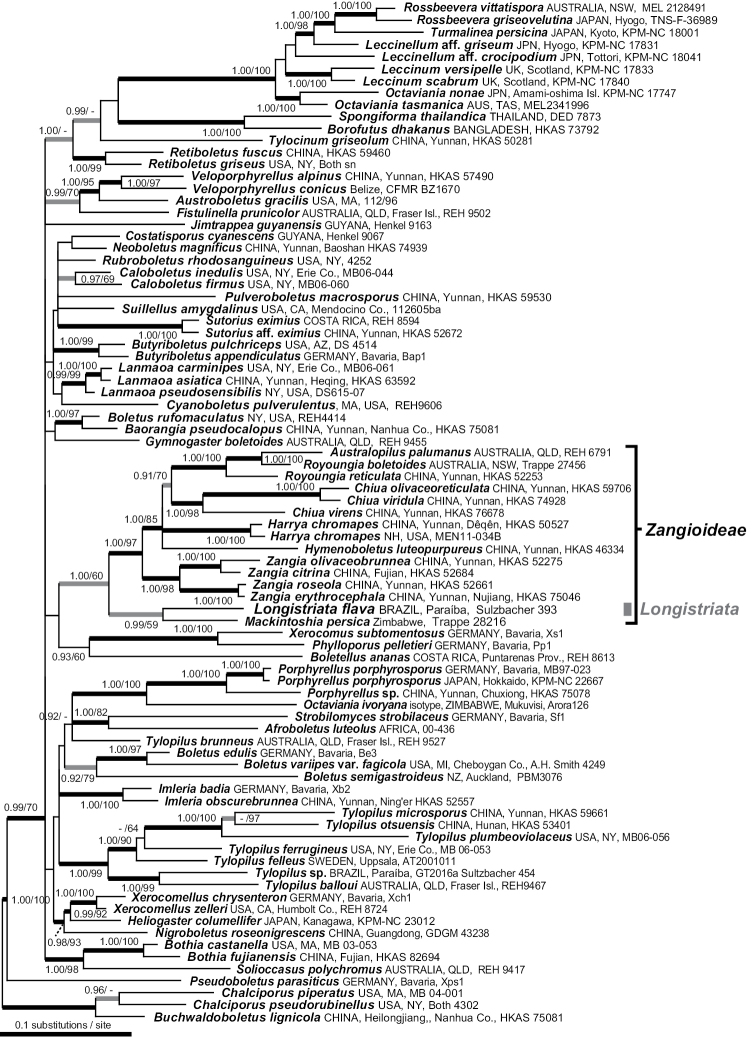
A *nLSU* + *TEF1* combined Maximum likelihood (ML) phylogram showing the phylogenetic relationship of *Longistriata* (UFRN-Fungus 1756, holotype) in relation to representative taxa in the Boletaceae. Non-parametric bootstrap branch supports (MPbs / MLbs) are given for nodes with bs>50.

The *nLSU + TEF1* combined tree of the Boletaceae supported our hypothesis that the sequestrate basidiomes of the vivid yellow fungus belong to an undescribed genus in the Boletaceae (Fig. 2). The species described here as *Longistriataflava* Sulzbacher, Orihara, Grebenc, M.P. Martín & Baseia, sp. nov. formed a sister lineage to the African monotypic sequestrate genus *Mackintoshia* (KC905034) with moderate to high statistical support (PP = 1.0, ML-BS = 59%). The phylogenetic analyses further suggested that the *Longistriata*-*Mackintoshia* clade is the earliest diverging lineage within the subfamily *Zangioideae* (PP = 1.0, ML-BS =60%). The epigeous yellowish bolete species (*Tylopilus* sp. Sulzbacher 454 in Suppl. material 1: Table S1) that sometimes occurred sympatrically with *Longistriataflava* was distantly related to *L.flava* and was instead more closely related to *Tylopilusballoui*. Other genera of Boletaceae that are closely related to *Longistriata* based on our phylogenetic analysis are species of *Australopilus* Halling & Fechner, *Chiua* Yan C. Li & Zhu L. Yang, *Harrya* Halling, Nuhn & Osmudson, *Hymenoboletus* Yan C. Li & Zhu L. Yang, *Royoungia* Castellano, Trappe & Malajczuk, and *Zangia* Yan C. Li & Zhu L. Yang. All sister clades have significant bootstrap support in phylogenetic analyses and show a range of morphological differences that support the erection of *Longstriata* as a separate genus. The ITS rDNA barcode sequences of *L.flava* specimens UFRN-fungos 1756 and UFRN-fungos 2110 were 751 bp in length (Suppl. material 1: Table S1). These sequences were less than 93% similar to all other ITS rDNA sequences in the INSD database. Below we describe this new genus and species and provide detailed morphological analysis and direct comparison with previously described sequestrate Boletaceae.

## Taxonomy

### Longistriata

Taxon classificationFungiBoletalesBoletaceae

Sulzbacher, Orihara, Grebenc, M.P. Martín & Baseia gen. nov.

3B5DE725-5CC2-5754-94A6-E8410CCFEF96

816322

#### Etymology.

*Longis* (Latin), with or from the long; *striatus* (Latin), striate, fluted; in reference to the distinctive series of thin longitudinal striations on the surface of the basidiospores.

#### Diagnosis.

**Figure 3.**
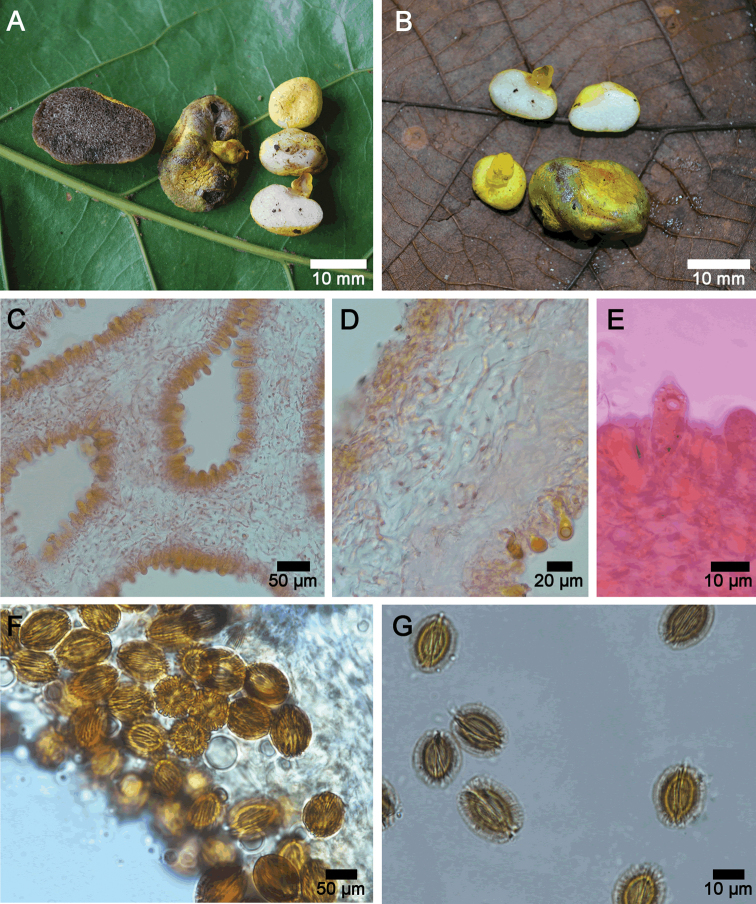
**A–G***Longistriataflava* (UFRN-Fungus1756, holotype) **A–B** fresh mature basidioma **C** hymenophoral trama mounted in 3% KOH with Congo Red **D** interwoven hyphae of peridium (upper left) and hymenophoral trama mounted in 3% KOH with Congo Red **E** hymenial cystidia mounted in 3% KOH with Congo Red **F** basidiospores mounted in Melzer’s reagent **G** basidiospores mounted in 3% KOH.

Distinguished from other genera in *Boletaceae* by a combination of the following characters: Basidiomata hypogeous to subhypogeous, sequestrate, subglobose, with a short stipe (Fig. 3A–B). Peridium bright yellow, smooth, with a cutis of interwoven and gelatinized inflated hyphae. Subgelatinous sterile base (a short stipe) present. Gleba loculate, white when immature to yellowish brown at maturity, turning dark green to black when cut in older basidioma, columella absent. Basidiospores broadly ellipsoid, hyaline to light brown at maturity, dextrinoid, with a series of thin, irregular longitudinal ridges across the spore surface; in some places these ridges are fused together. Cystidia are lageniform with rounded apices. Clamp connections absent. Found in white sand habitat in tropical ectotrophic forests. Potentially mycorrhizal with tropical ectomycorrhizal plants from genera *Coccoloba* (Polygonaceae) and *Guapira* (Nyctaginaceae).

#### Type species.

*Longistriataflava* Sulzbacher, Orihara, Grebenc, M.P. Martín & Baseia, sp. nov.

### Longistriata flava

Taxon classificationFungiBoletalesBoletaceae

Sulzbacher, Orihara, Grebenc, M.P. Martín & Baseia sp. nov.

A0845E6B-1CDF-5CB3-A7D1-A0F4785D6D50

816323

Figs 3 , 4 , 5

#### Etymology.

*Flavus* (Latin), refers to the yellow peridium of the species.

**Holotype**: Brazil, Paraíba State, Mamanguape, Guaribas Biological Reserve, 06°44.545'S, 35°08.535'W, 14.VII.2012, leg. *Sulzbacher–393* (UFRN-fungos 1756). GenBank accession number for ITS, nLSU and *TEF1*: LT574840; LT574842; LT574844

#### Description.

Basidiomata hypogeous to subhypogeous, 11–24 mm wide, 13–16 mm high; subglobose, depressed subglobose to oblong in older stages, with small folds at the base; with a short stipe (Fig. 3A–B). Peridium <0.8 mm thick, at younger stages yellow (2A6) to light yellow (1A5) then yellowish brown (5D8) to brownish yellow (5C8) at maturity; smooth and glabrous, sometimes finely fibrillose. Sterile base present, short, 6–8 × 3–4 mm, clavate with a bulbous slightly developed base; color vivid yellow (3A8), brownish yellow (5C8) when bruised; surface glabrous, with small folds and depressions; the inner part is full, subgelatinous and yellowish brown (5D8); connected by scattered and short, thin (0.3–0.5 mm diam), orange (6B8) rhizomorphs. Gleba loculate, non-gelatinized to gelatinized, with irregular locules (0.5–1 mm diam); white (1A1) at younger stages, to finally yellowish brown (5F4) at maturity, immediately turning deep green (30F7) to black when cut in older basidiomata.

**Figure 4.**
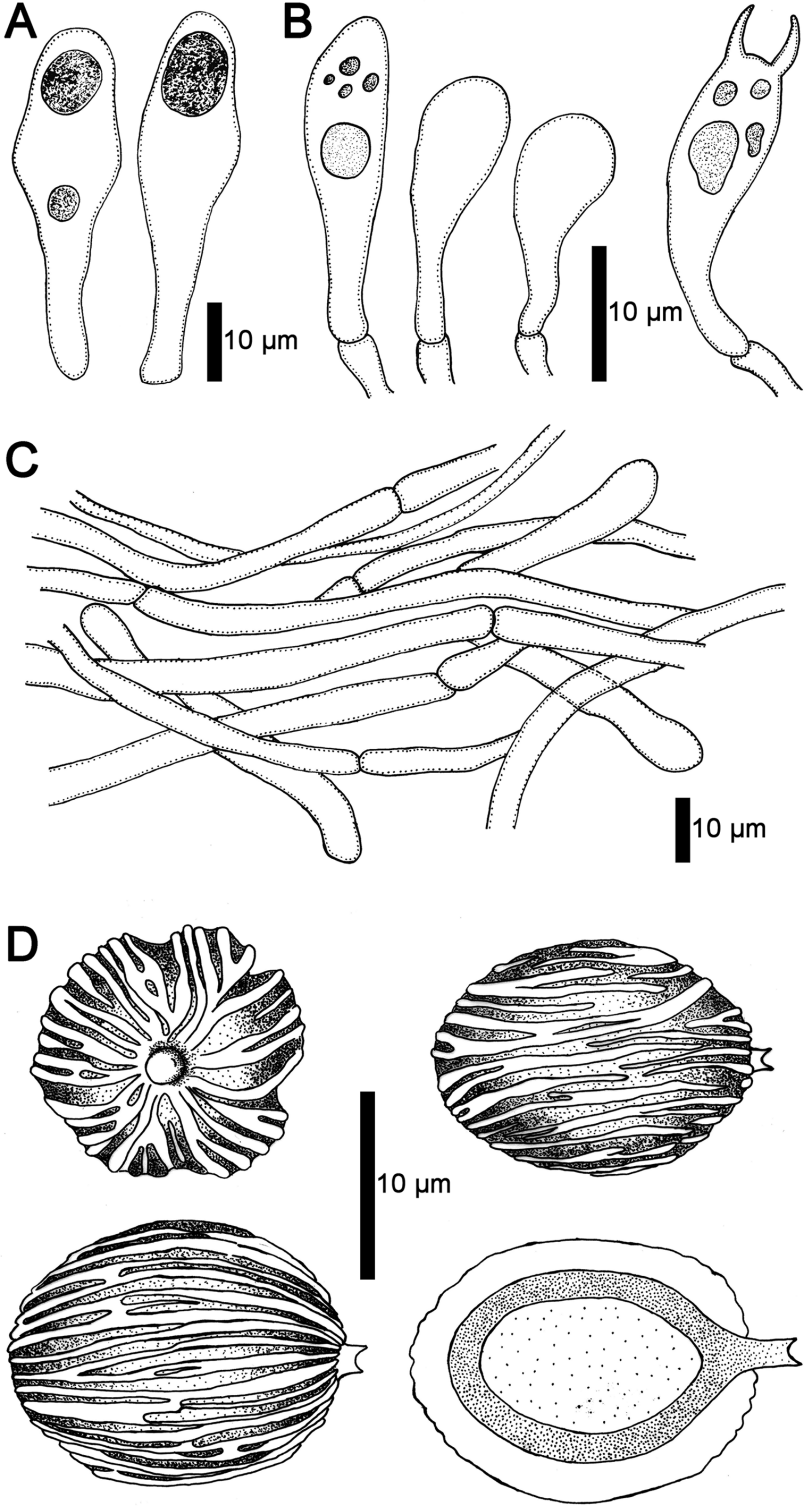
**A–D***Longistriataflava* (UFRN-Fungus 1756, holotype) **A** hymenial cystidia **B** basidioles and basidium **C** details of the peridium with interwoven hyphae **D** polar and longitudinal view of basidiospores.

**Figure 5.**
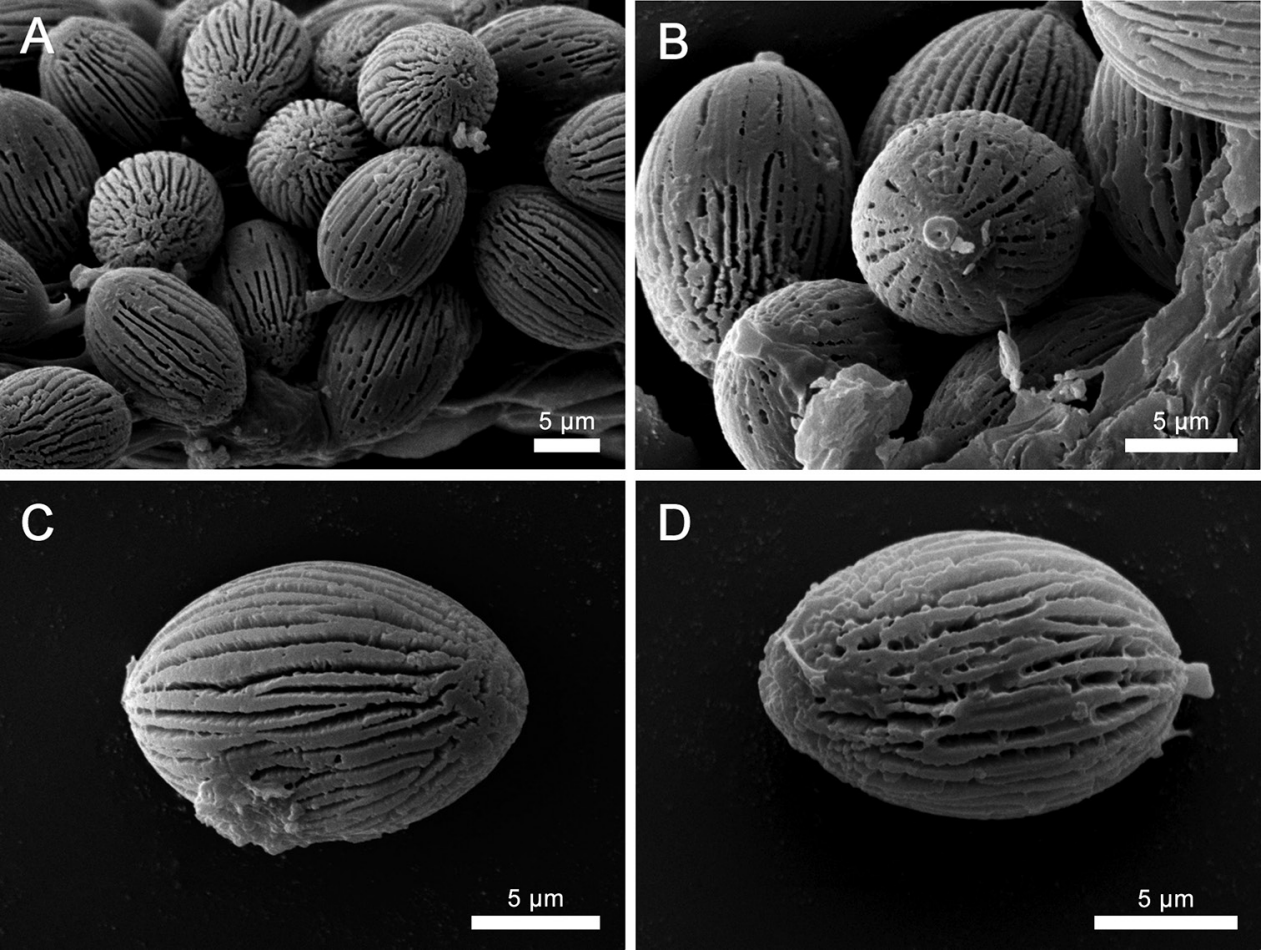
**A–D** Basidiospores of *Longistriataflava* (UFRN-Fungus 1756, holotype) as observed with scanning electron microscopy. Note the persistent sterigmal attachment and a series of thinner longitudinal ridges (on average > 10 complete ridges across the longitudinal axis of the spore) with additional irregular, thin, low and bifurcated or fused ridges.

Peridium 100–200 µm thick, composed by a cutis of interwoven hyphae and immersed in a gelatinized matrix (Fig. 4C), 2–6 µm diam., with rounded, thin-walled, smooth, terminal hyphae, not readily separable from gleba. Hymenophoral trama formed by parallel to subparallel, smooth and thin-walled, hyaline hyphae, inamyloid, gelatinized in the central part, 3–6 µm diam (Fig. 3C–D). Subhymenium ramose, 46–72 µm deep, hyphae 10–16 × 2–5 µm diam. Hymenial cystidia 38–78.5 × 10–14 μm, lageniform or ventricose, with rounded apex, thin-walled, hyaline, inamyloid (Figs 3E, 4A). Basidia 25–48 × 10–15 µm, clavate, 2 and 4-spored (sterigmata up to 3 µm long.), hyaline. Basidioles 31–46 × 7–12 μm, clavate with rounded apex (Fig. 4B). Basidiospores [30/2/2] 15–19 (–20) × 13–16 (–17) µm (ornamentation included), [L = 17.7 μm, W = 14.7 μm, Q = 1.10–1.40 (–1.50), Qm = 1.20], broadly ellipsoid, sterigmal attachment persistent at maturity, up to 3 µm long; hyaline when young to finally light brown at maturity in 3% KOH, dextrinoid in Melzer’s reagent; walls ornamented (< 2.5 µm width); with a series of thinner longitudinal ridges, in average > 10 complete ridges across the longitudinal axis of the spore with additional irregular, thin and low ridges that are sometimes bifurcated (Figs 3F–G, 4D) or fused together (Fig. 5C); under a scanning electron microscope the surface is clearly longitudinally striated (Fig. 5A–D).

#### Habitat.

Hypogeous to subhypogeous, solitary or scattered, under fallen leaves or in O1 soil horizon, in sandy soil, among trees in Brazil’s Atlantic rainforest, in vicinity of *Coccolobaalnifolia* Casar., *C.laevis* Casar. (Polygonaceae) and species of *Guapira* Aubl. (Nyctaginaceae). Species in both plant genera (*Coccoloba* and *Guapira*) have been consistently confirmed as ectomycorrhizal hosts throughout the Neotropics (Tedersoo et al. 2010b). All known specimens were found in silicate silt to sandy soils, with moderately low pH (4.5–5.5), low available nutrients and low water capacity. Despite the close vicinity of the ocean, the absence of halophilic vegetation indicates a lack of salinification or accumulation of NaCl in soils.

#### Distribution.

Known only from the type locality.

#### Additional specimens examined.

Brazil, Paraíba State, Mamanguape, Guaribas Biological Reserve, 06°44.545'S, 35°08.535'W, 27.VII.2012, leg. *Sulzbacher–466* (paratype UFRN-fungos 2110, LJF 1203). GenBank accession number for ITS: LT574839.

#### Additional Comments.

The specimens UFRN-fungos 1756 and UFRN-fungos 2110 are sequestrate to emergent basidiomes that fruit in small groups. The basidiomes have a smooth and vivid yellow peridium that becomes dark green when exposed to air. They also have a central sterile base that is attached to short orange rhizomorphs, a white gleba formed of distinct locules that turns dark green to black when cut and hyaline to light brown, broadly ellipsoid basidiospores covered with a series of thin, dextrinoid longitudinal striations and ridges. These ridges and striations are sometimes bifurcated or irregular and they also cover the entire spore surface. The clavate basidia can be either 2-spored and 4-spored and the lageniform to ventricose cystidia are a notable feature in the hymenium. This combination of morphological features is unique within the Boletaceae.

## Discussion

*Longistriata* is a striking new monotypic genus described from the Atlantic forest in the Northeastern part of Brazil. The only known representative of the genus is the newly described *Longistriataflava*. This species is characterized by the hypogeous habit, a smooth and bright yellow peridium (Fig. 3), presence of cystidia, and the absence of clamp connections in all tissues (Fig. 4). Based on a combined phylogenetic analysis of *nLSU* + *TEF1* the closest relative is *Mackintoshiapersica* (Fig. 2). However, *L.flava* is differentiated from *M.persica* (Pacioni and Sharp 2000) based on its well-developed, sterile base that forms a short stipe, lageniform cystidia with rounded apices, basidiospores with persistent sterigmal attachments that are covered by thin longitudinal striations and spores that are 15–19 × 13–16 µm. *Mackintoshia* has smooth and smaller elliptical basidiospores (8–12 × 5–7 μm) (Pacioni and Sharp 2000) and is known only from Africa (Castellano et al. 2000). The two genera also have different host plants; *Mackintoshia* is found in habitats dominated by ECM plants in the Fabaceae and Uapacaceae (Pacioni and Sharp 2000) whereas *Longistriata* is found with ECM plants in Nyctaginaceae and Polygonaceae. This combination of morphological features is unique, separating the sister clade *Mackintoshia* from *Longistriata*.

In addition to *Longistriata* several other genera of sequestrate Boletaceae, *Chamonixia*, *Rossbeevera*, *Rhodactina*, and *Turmalinea*, also have basidiospores with longitudinal ridges. However, members of these genera all differ in the shape and number of ridges. *Rosbeevera* has ellipsoid to fusiform basidiospores with 3–5 ridges (Lebel et al. 2012), *Chamonixia* has subglobose to broadly ellipsoid basidiospores with 6–10 ridges (Lebel et al. 2012), *Rhodactina* has broadly ellipsoid to subfusiform basidiospores with 8–10 ridges (Yang et al. 2006), and *Turmalinea* has ovoid to fusoid basidiospores with 5–10 longitudinal ridges that are often branched to irregularly broken and spores can be with or without a hilar appendage (Orihara et al. 2016b). The spore colors are also different in these other genera; *Rossbeevera* and *Chamonixia* have brown to brownish spores (Montecchi and Sarasini 2000; Lebel et al. 2012), *Turmelinea* has inamyloid, non-dextrinoid, spores that are brick red to dark brown at maturity (Orihara et al. 2016b) and *Rhodactina* species have spores that are deep purple (Yang et al. 2006). In *Longistriata* the number of ridges is greater than in any of the other genera. On average, spores of *Longistriataflava* have 10 complete ridges across the longitudinal axis of the spore with additional irregular, thin and low ridges (Figs 3F–G, 4D). Furthermore, the ridges in this species are thin, low and irregular as compared to the other genera listed above. In some spores the ridges of *Longistriata* can be fused or bifurcating (Fig. 5C). Species in the sequestrate genus *Gautieria* also has spores with longitudinal ridges but this genus is very different from *Longistriata* because the basidioma of *Gautieria* species often lack a peridium and they belong in the distantly related order Gomphales (Montecchi and Sarasini 2000; Giachini et al. 2010). The ridged basidiospores of *Longistriata* are also superficially similar to those of the epigeous bolete genus *Boletellus* because species in both genera typically have longitudinal ridges. However, our phylogenetic analyses indicate that these two genera are only distantly related within the Boletaceae (Fig. 2).

The hypogeous habit, shape of basidiomes (e.g. globose, subglobose, tuberiform) and the rudimentary sterile base in *L.flava* suggest a possible relationship with the sequestrate truffle-like genus *Octaviania* (Orihara et al. 2012). However, the basidiospores are very different in *Octaviania* (e.g. globose to ellipsoid spores with ornamentation of large, thick-walled, pyramidal to conical ornaments) and molecular data indicate that *Octaviania* is a distant relative of *Longistriata*. The bright yellow peridium of fresh basidiomata and the presence of a stipe in *L.flava* resembles members of the *Boletuschromapes* group (e.g. *Zangia* and *Harrya*) as well as the genus *Royoungia* where at least some taxa have similar bright yellow coloration (Li et al. 2011; Halling et al. 2012).

The phylogenetic analyses suggest that the new Brazilian genus is closely related to several genera in the subfamily Zangioideae that also have bright yellow colors at the base of the stipe (e.g. *Chiua* Yan C. Li & Zhu L. Yang, *Harrya* Halling, Nuhn & Osmudson, *Royoungia* Castellano, Trappe & Malajczuk, and *Zangia* Yan C. Li & Zhu L. Yang) (Wu et al. 2014, 2016) (Fig. 2). Within the Zangioideae only one hypogeous sequestrate taxon, *Royoungiaboletoides*, was previously known (Wu et al. 2014, 2016). All of the other genera in Zangioideae are characterized by the epigeous habit, with a well-developed and central stipe and smooth basidiospores. The fresh appearance of *Longistriataflava*, with its bright yellow peridium, resembles the colors found in *Chiua* or *Zangia* (from Asia with Fagaceae and Pinaceae) or *Royoungia* (from Australia with Myrtaceae) (Li et al. 2011; Halling et al. 2012; Wu et al. 2016).

Several other sequestrate Boletaceae are similar to *L.flava*, either in their morphology or in their tropical distribution. Members of the sequestrate genus *Mycoamaranthus* Castellano, Trappe & Malajczuk also produce bright yellow basidiomata and belong to Boletaceae (Binder and Hibbett 2006) but GenBank BLASTn queries based on the ITS rDNA indicate that *Longistriata* is distantly related to *Mycoamaranthus* (e.g. the ITS is <85% similar to both *Mycoamaranthuscongolensis* and *M.cambodgensis*). Another genus that shares several morphological similarities with *Longistriata* is the genus *Solioccasus* (Trappe et al. 2013). This genus differs from *Longistriata* by the large and copious rhizomorphs appressed to peridial surfaces, a dendroid and cartilaginous columella, smooth basidiospores, and basidiomes with bright orange and reddish coloration. The monotypic genus *Afrocastellanoa* from tropical Africa (Orihara and Smith 2017) is distinct from *Longistriata* because it is characterized by whitish basidiomata, globose to subglobose basidiospores with warty to spiny spore ornaments and its phylogenetic relationship with the epigeous genus *Porphyrellus*. Recently, Smith et al. (2015) discovered three new monotypic hypogeous sequestrate genera within Boletaceae, *Jimtrappeaguyanensis* T.W. Henkel, M.E. Smith & Aime, *Castellaneapakaraimophila* T.W. Henkel & M.E. Smith and *Costatisporuscyanescens* T.W. Henkel & M.E. Smith (Smith et al. 2015). Like *Longistriata*, all three new genera are endemic to tropical South America but their macro- and microscopic characteristics are notably different. *Jimtrappeaguyanensis* is characterized by the white peridium, unchanging tissues, short columella, smooth subfusiform, reddish brown basidiospores and prominent dextrinoid cystidia (Smith et al. 2015). The white peridium, the unchanging tissues, smooth subfusiform basidiospores, and prominent cystidia of *J.guyanensis* contrast with the yellow peridium and unique basidiospores morphology of *L.flava*. Phylogenetic analysis also confirms that *J.guyanensis* and *L.flava* are not closely related. *Castellaneapakaraimophila* is similar to *L.flava* because both have subglobose basidioma and a short stipe but *L.flava* has a bright yellow peridium. The two species can also be easily differentiated by their spores because *C.pakaraimophila* has smooth, subfusiform basidiospores whereas *L.flava* has ellipsoid spores with distinct longitudinal striations (Smith et al. 2015). *Costatisporuscyanescens* is easily differentiated from *L.flava* by its grayish yellow peridium and the dark blue staining reaction on the peridium. Microscopically, the longitudinally ridged basidiospore ornamentation of *C.cyanescens* (fig. 4c, in Smith et al. 2015) is similar to that in *L.flava* (Fig. 5A–D). However, the basidiospores are broadly ellipsoid and dextrinoid in *L.flava* and the ornamentation is formed by a series of thin longitudinal striations on all surfaces. In contrast, the spores of *C.cyanescens* are unreactive in Melzer’s reagent and are ovate to subfusiform with ridges that are somewhat spiraled. The two species are also distantly related based on our phylogenetic analysis (Fig. 2). The ecology of *Jimtrappea*, *Castellanea* and *Costatisporus* are also different from *Longistriata*. These three genera are apparently endemic to the Guiana Shield and are associated with the ECM tree genera *Aldina*, *Dicymbe* and *Pakaraimaea* (Smith et al. 2015).

Unfortunately, we have not yet confirmed the ECM status of *Longistriataflava* based on sequences from ECM root tips from native Brazilian trees. However, basidiomes of *Longistriata* have always been collected in the lowland semi-deciduous forest to savanna known as “tabuleiro” in close proximity to woody plants in the ECM genera *Coccoloba* (Polygonaceae) and *Guapira* (Nyctaginaceae). Given that ECM plants in these genera are known to host a wide array of ECM fungi from other sites in tropical South America (Tedersoo et al. 2010; Séne et al. 2015; Põlme et al. 2017) and that other taxa in the Zangioideae are known to be ECM (Tedersoo and Smith 2013), we hypothesize that *L.flava* is also ECM. The ECM nutritional mode is also likely to be favored in the nutrient-poor sandy soil ecosystem of Neotropical forest fragments of the Atlantic Forests.

## Supplementary Material

XML Treatment for Longistriata

XML Treatment for Longistriata flava

## Supplementary materials

Supplementary material 1 Table S1. Specimens and sequences used for the molecular phylogenetic analyses (i. e., the nLSU + TEF1 combined dataset) and ITS sequence comparison. Data type: molecular data https://binary.pensoft.net/file/375569This dataset is made available under the Open Database License (http://opendatacommons.org/licenses/odbl/1.0/). The Open Database License (ODbL) is a license agreement intended to allow users to freely share, modify, and use this Dataset while maintaining this same freedom for others, provided that the original source and author(s) are credited.Marcelo A. Sulzbacher, Takamichi Orihara, Tine Grebenc, Felipe Wartchow, Matthew E. Smith, María P. Martín, Admir J. Giachini, Iuri G. Baseia
